# Gentiopicroside ameliorates the lipopolysaccharide-induced inflammatory response and hypertrophy in chondrocytes

**DOI:** 10.1186/s13018-024-04676-1

**Published:** 2024-03-25

**Authors:** Longfei Li, Qianqian Fan, Yixuan Zhao, Qian Zhang, Gaofeng Qin, Chen Li, Wei Li

**Affiliations:** 1 School of Special Education and Rehabilitation, Binzhou Medical University, Yantai, Shandong China; 2 Department of Rehabilitation, Binzhou Medical University Hospital, Binzhou, Shandong China

**Keywords:** Osteoarthritis, Knee osteoarthritis, Gentiopicroside, Lipopolysaccharide, Hypertrophy

## Abstract

**Purpose:**

This study aimed to evaluate the protective effects of gentiopicroside against lipopolysaccharide-induced chondrocyte inflammation.

**Methods:**

SW 1353 chondrosarcoma cells were stimulated with LPS (5 μg/ml) for 24 h and treated with different concentrations of gentiopicroside (GPS) for 24 h. The toxic effects of GPS on chondrocytes were determined using a CCK-8 assay and EdU staining. Western blotting, qPCR, and immunofluorescence analysis were used to examine the protective effect of GPS against the inflammatory response in chondrocytes induced by lipopolysaccharide (LPS). One-way ANOVA was used to compare the differences between the groups (significance level of 0.05).

**Results:**

The CCK-8 results showed that 10, 20 and 40 μM GPS had no significant toxic effects on chondrocytes; GPS effectively reduced the production of IL-1β and PGE2, reversed LPS-induced extracellular matrix degradation in cartilage by inhibiting the Stat3/Runx2 signaling pathway, and suppressed the hypertrophic transformation of SW 1353 chondrosarcoma cells.

**Conclusion:**

Our study demonstrated that GPS significantly inhibited the LPS-induced inflammatory response and hypertrophic cellular degeneration in SW 1353 chondrosarcoma cells and is a valuable traditional Chinese medicine for the treatment of knee osteoarthritis.

## Introduction

Knee osteoarthritis (KOA) is a chronic disease that is often accompanied by chronic pain and stiffness [1] and is highly prevalent in elderly individuals, obese individuals, and those with a history of knee trauma [2]. Long-lasting chronic pain and the resulting emotional disorders seriously affect patient quality of life, and the long treatment cycle and accompanying high treatment costs increase the economic burdens on patients and society [3]. At present, more than 22% of adults over the age of 40 worldwide have KOA [4]. The pathological changes associated with KOA include articular cartilage destruction, subchondral bone remodeling, and synovial inflammation [5, 6]. The destruction of articular cartilage is considered the main pathological change in KOA, which is characterized by excessive degradation of the extracellular matrix and chondrocyte degeneration [7]. Chondrocytes are the only cells in knee cartilage tissue. The extracellular matrix (ECM), which is mainly composed of collagen II, is synthesized and secreted to maintain normal cartilage shape and balance stress in the knee joint [8, 9] and has important clinical importance in the pathogenesis of KOA. Therefore, an in vitro model simulating chondrocyte degeneration would be valuable for exploring the pathogenesis and treatment of KOA.

lipopolysaccharide (LPS) is a polysaccharide compound that can be used as a pathogenic factor and causes an inflammatory response in the body and abnormal activation of the immune system [10]. Local injection of LPS induces synovitis and inflammatory cartilage damage, and an LPS-induced chondrocyte inflammation model effectively reflects the extent of OA cartilage damage [11]. The SW 1353 cell line, which is derived from chondrosarcoma, is a commonly used in vitro model for studying osteoarthritis [12]; therefore, in this study, we used these cells to simulate the inflammatory response and catabolism associated with OA in response to LPS stimulation.

Treatment of KOA includes health management, medication and surgery [13]. Due to the lack of vascular and lymphatic tissues and its limited proliferative capacity, cartilage tissue is difficult to repair after injury or degeneration [14]. Conventional treatments can only alleviate clinical symptoms and cannot repair damaged cartilage tissue or slow disease progression [15]. In recent years, herbal monomers derived from plants have been the first choice for treating inflammatory diseases [16]. Gentiopicroside (GPS) is a natural iridoid glycoside compound extracted from gentian that has anti-inflammatory, analgesic, antioxidant, and other biological activities [17]. GPS can significantly inhibit the expression of marker genes related to osteoclast generation in mouse bone marrow macrophages stimulated with an NF-κB ligand receptor activator, inhibit osteoclast generation, and ameliorate the symptoms of osteoporosis in mice [18]. Studies have shown that GPS can alleviate synovitis and cartilage destruction in mice with collagen-induced rheumatoid arthritis and exerts antirheumatic effects and protects cartilage by decreasing the secretion of matrix metalloproteinases (MMPs), suggesting that GPS is a potential drug for the treatment of rheumatoid arthritis [19].

Kindlin-2 is a key adhesion protein that interacts with the cytoplasmic domain of integrin and is highly expressed in healthy chondrocytes [20]. In OA chondrocytes, a low level of Kindlin-2 promotes inflammation and catabolism through the activation of Stat3 phosphorylation [21]. There have been numerous studies on the anti-inflammatory effects of GPS [18, 19]; however, whether GPS ameliorates inflammation and chondrocyte hypertrophy in the LPS-induced SW 1353 chondrosarcoma cells via the Stat3/Runx2 signaling pathway has not been reported. Therefore, we evaluated the effects of GPS in a LPS-induced KOA cartilage model using cellular experiments and examined the molecular mechanisms by which the Stat3/Runx2 signaling pathway mediated the therapeutic effects of GPS.

## Materials and reagents

The following materials and reagents were used: SW 1353 cells (Pricella, China), GPS (MCE, China), LPS, toluidine blue stain (Sigma, USA), fetal bovine serum (Corning, USA), DMEM, high-sugar medium, green streptomycin double resistance solution (Servicebio, China), CCK-8 kits, and IL-1β, IL-10, and PGE2 enzyme-linked immunosorbent assay kits (Solarbio, China). 5-Ethynyl-2'-deoxyuridine (EdU)-488 cell proliferation detection kits (Beyotime, China). COX-2, IL-β, IL-10 antibodies (Affinity, China), ADAMTS5, MMP-13, col2, ACAN and col10 antibodies (Proteintech, China) were used. Kindlin-2, Runx2, Stat3, and P-Stat3 antibodies (Abcam, UK), TRIzol reagent (Invitrogen, USA), PCR kits (Vazyme, China), and fluorescent quantitative PCR primers (Sangon, China) were used.

## Methods

### Identification of chondrocytes

Cells were seeded in 6-well plates at a density of 1 × 10^4^/well, covered with polylysine slides, and incubated at 37 °C for 24 h. Then, the prepared cells were fixed with 4% paraformaldehyde for 15 min, rinsed with phosphate-buffered saline (PBS) 3 times, and stained with 1% toluidine blue solution for 30 min. After the cells were rinsed twice with PBS, cell morphology was observed using an inverted fluorescence microscope [22].

### Cell viability assessment

The toxic effects of GPS on cells were examined using a CCK-8 kit [23]. Chondrocytes were seeded in 96-well plates at a density of 3 × 10^3^ cells/well. Once the cell density reached 80%, 0, 10, 20, 40, 80, or 160 μM GPS was added and incubated for 24 h. The medium was replaced, 10 μL of CCK-8 solution was added to each well, the plates were shaken until mixed, and the plates were then placed in an incubator at 37 °C for 4 h. The OD value at 450 nm was determined using a multifunctional enzyme standard reader (blank group without cells; control group without GPS). To examine the effect of GPS on the proliferation of osteoarthritic chondrocytes, the cells were first pretreated with 5 μg/ml LPS for 24 h [24]. Then, 10, 20 and 40 μM GPS was added and incubated for 24, 48 and 72 h to examine effects of different concentrations of GPS on the proliferation of osteoarthritic chondrocytes.

### EdU staining

Cells were inoculated into 12-well plates at a density of 5 × 10^3^ cells/well, pretreated with 5 μg/ml LPS for 24 h, and then treated with 0, 10, 20, or 40 μM serum-free GPS solution for 24 h. One milliliter of EdU working solution (20 μM) was added to each well and incubated for 2 h. The cells were fixed with 4% paraformaldehyde for 15 min and permeabilized with 0.3% Triton X-100 for 15 min. Finally, the cells were incubated with 200 μl of click reaction solution for 1 h. The nuclei were stained with Hoechst 33,342 for 10 min [25]. Chondrocyte proliferation was observed using an inverted fluorescence microscope, and the percentage of positive cells was calculated.

### Cell groupings and interventions

According to the previous results, the cells were divided into the control group (G0), model group (G1), LPS + GPS low-dose group (G2), LPS + GPS medium-dose group (G3), and LPS + GPS high-dose group (G4). Cells in each group (except the control group) were treated with 5 μg/ml LPS to establish the osteoarthritis cell model and then treated with 10, 20, or 40 μM GPS [24, 26].

### Measurement of IL-1β and PGE2

The cells were treated as described above, and the supernatant was collected. The levels of the inflammatory factors IL-1β and PGE2 in the culture medium were examined by ELISA [27].

### Immunofluorescence staining

In each group, cell crawlers were prepared, fixed in 4% paraformaldehyde for 15 min, permeabilized with 0.5% Triton-X-100 for 15 min, rinsed with PBS, blocked with 5% bovine serum albumin (BSA) for 1 h at room temperature, rinsed twice with PBS, and incubated with a col2 antibody at 4 °C overnight. On the second day, a fluorescence-conjugated secondary antibody was added and incubated in the dark for 1 h. The cells were washed 3 times with PBS and then stained with DAPI for 10 min. The slices were then blocked, and images of col2 were captured by confocal laser scanning microscopy [28].

### Western blot analysis

Cells in each group were collected and placed on ice for 30 min in RIPA lysis buffer mixed with PMSF. Total protein was extracted, and the protein concentration was determined using a BCA protein assay kit. Equal amounts of proteins were separated by SDS‒PAGE, transferred to PVDF membranes, sealed with protein-free rapid sealing solution for 10 min, incubated with primary antibody at 4 °C overnight after being washed with TBST, and then incubated with specific secondary antibodies at room temperature for 1 h. After being washed three times with TBST, the membrane was placed in a chemiluminescent color-developing solution in the dark, and statistical analysis was performed [29].

### Real-time quantitative PCR

Cells in each group were collected, total RNA was extracted by an RNA isolation and extraction kit, the RNA was reverse transcribed into cDNA by a reverse transcription kit, the housekeeping gene GAPDH was selected as an internal reference, and real-time fluorescence quantitative PCR was performed by using SYBR® GreenERTM SuperMix Universal (Invitrogen®) [30]. Relative expression was calculated by the 2-ΔΔCt method (ΔCt is the difference between the threshold cycle of the samples and the reference value of GAPDH), and the sequences of the primers used in the experiment are listed in Table 1.

**Table 1:** Primer sequences of genes

Gene name	Base sequence 5′-3′	Length
Collagen II	F: GTAACCCTGGAACAGATGGAAT	149
	R: TTCACCCGTCTGACCTTTCG	
Collagen X	F: ACAGGCAACAGCATTATGACCC	198
	R: CGATGATGGCACTCCCTGAA	
Aggrecan	F: ATTTCAGCGGTTCCTTCTCCA	212
	R: GTATAGGCTGGTTCCCATTCTG	
MMP-13	F: AATGCAGTCTTTCTTCGGCTTAG	188
	R: CAGAATGAGTCATATCAGGGGTGT	
ADAMTS 5	F: GAGCCTGGAAGTGAGCAAGAA	137
	R: CACATAAATCCTCCCGAGTAAACA	
IL-1β	F: CGATCACTGAACTGCACGCTC	131
	R: ACAAAGGACATGGAGAACACCACTT	
COX2	F: AGCACTTCACGCATCAGTTTTTC	205
	R: GCCTGAGTATCTTTGACTGTGGG	
GADPH	F: GGAAGCTTGTCATCAATGGAAATC	168
	R: TGATGACCCTTTTGGCTCCC	

### Statistical analysis

SPSS 28.0.1.1 software was used to analyze the experimental results. Measurement data are expressed as the mean ± standard deviation (SD). One-way ANOVA was used to compare the differences between groups (significance level of 0.05).

## Results

### Toluidine blue staining

**Fig. 1:**
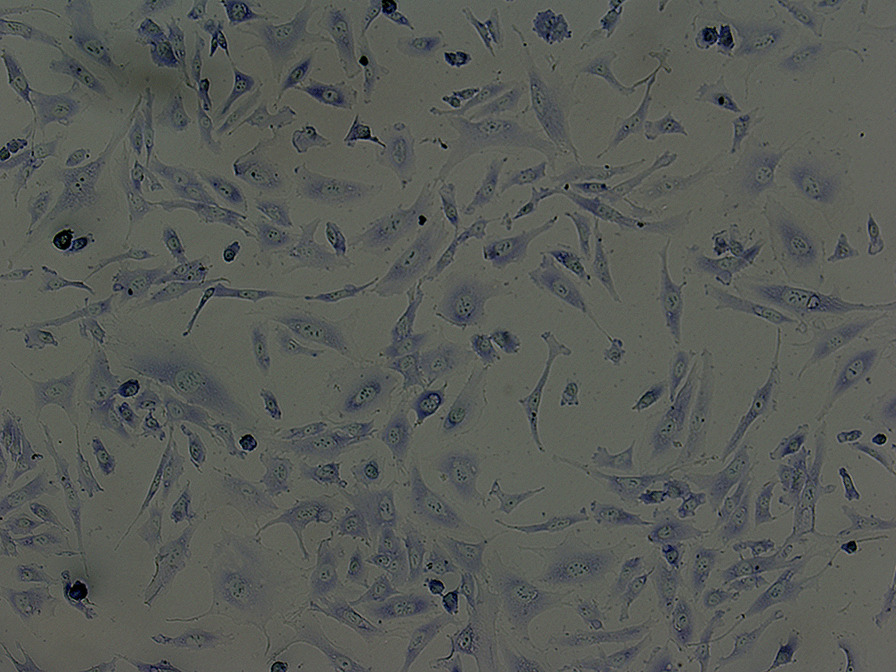
Results of toluidine blue staining of chondrocytes

Toluidine blue staining showed that the proteoglycan secreted by chondrocytes was heterochromatic when it combined with toluidine blue staining solution. The cells grew in monolayers, some were polygonal, and some were elongated; the cytoplasm was bluish-purple, verifying that the cultured cells were chondrocytes (Fig. 1).

### Effect of GPS on chondrocyte viability

**Fig. 2:**
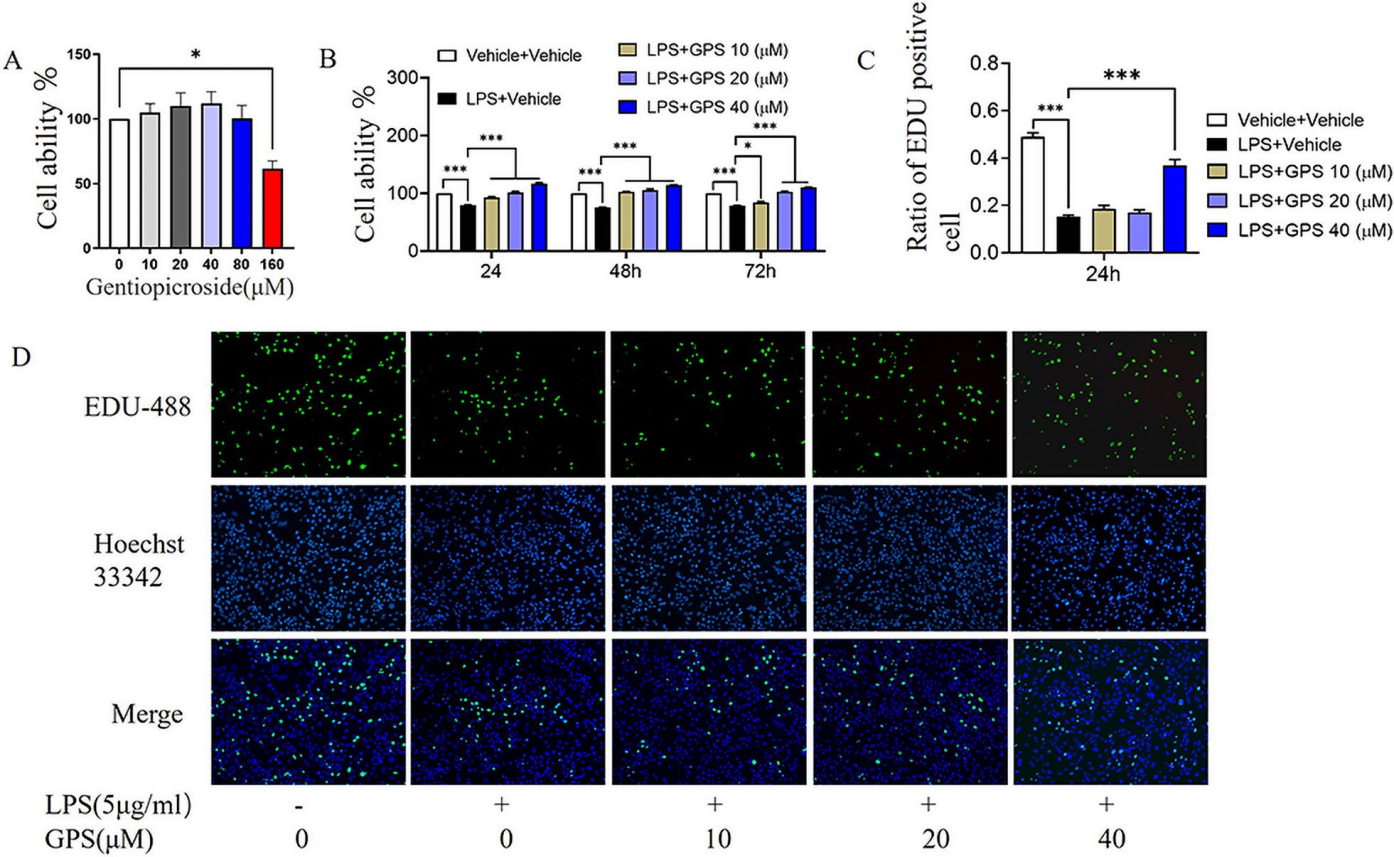
Effects of GPS on cell viability. **A** GPS (0, 10, 20, 40, 80 and 160 μM) was incubated with chondrocytes for 24 h. Cell viability was determined by a CCK-8 assay. **B** Chondrocytes were pretreated with 5 μg/ml LPS for 24 h and then treated with different concentrations of GPS (10, 20 and 40 μM) for 24, 48 and 72 h, and cell viability was determined by a CCK-8 assay. **C** Proportion of chondrocytes in the proliferative phase relative to the total cell number. **D** Chondrocytes were pretreated with 5 μg/ml LPS for 24 h and then treated with different concentrations of GPS (10, 20 and 40 μM) for 24 h. Proliferative chondrocytes were labeled with EdU; green cells indicate proliferating chondrocytes, and nuclei are depicted in blue. *P < 0.05, ***P < 0.001

The results showed that 0, 10, 20, 40, and 80 μM GPS had no toxic effects on chondrocytes. However, the growth of chondrocytes was inhibited by 80 μM GPS, and chondrocyte survival was significantly inhibited 160 μM GPS (Fig. 2A). Therefore, concentrations of 10, 20, and 40 μM were selected for subsequent experiments. The proliferation rate of chondrocytes significantly decreased after LPS exposure, and the LPS-induced decrease in the proliferation rate of chondrocytes was reversed by adding GPS to the culture medium (Fig. 2B). Chondrocyte apoptosis was significantly inhibited by 40 μM GPS, which significantly increased the proportion of proliferating chondrocytes among LPS-treated chondrocytes (Fig. 2C). EdU staining showed that GPS inhibited chondrocyte apoptosis after LPS treatment (Fig. 2D).

### Effect of GPS on LPS-induced chondrocyte inflammation

**Fig. 3:**
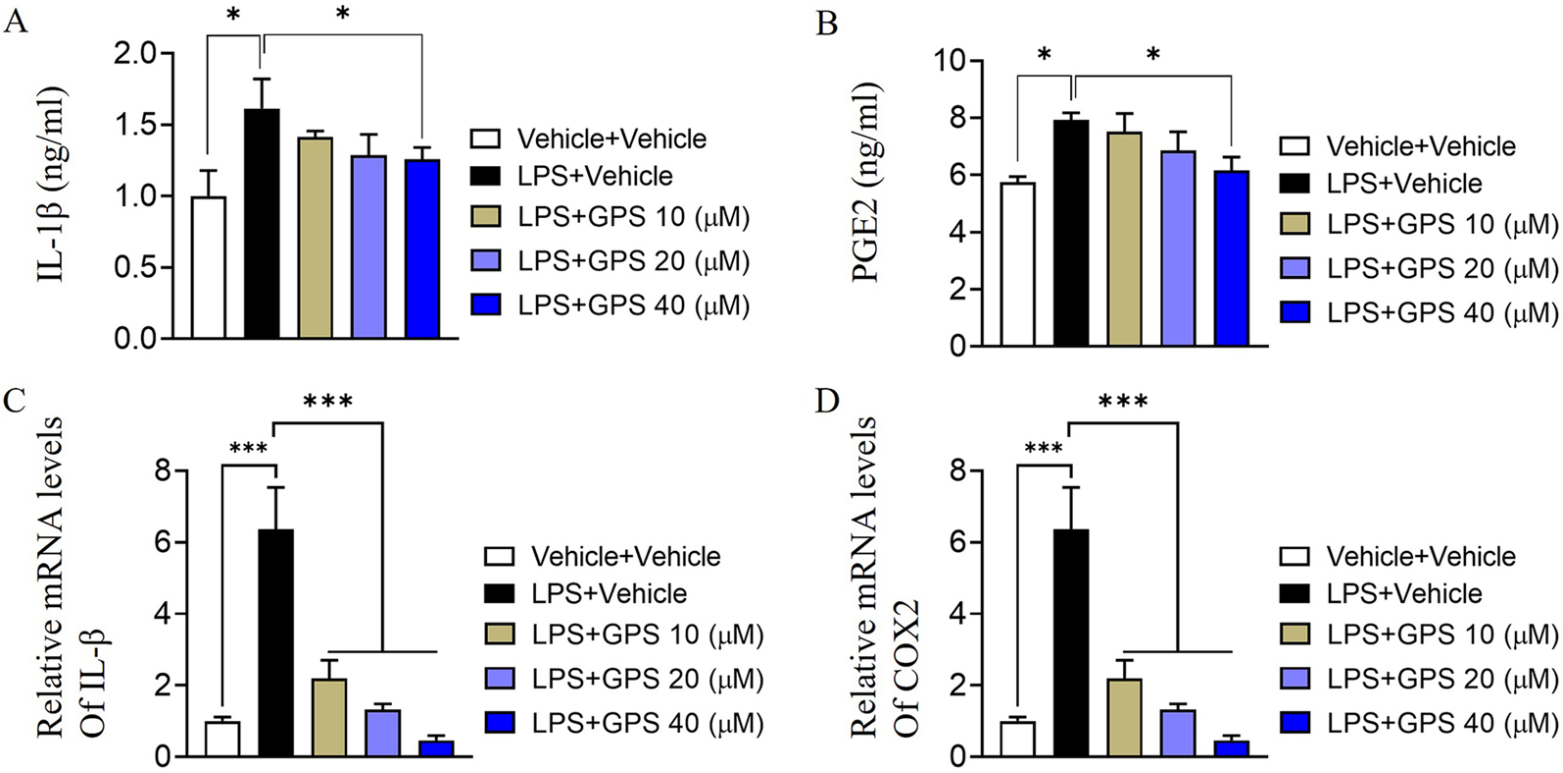
Inhibitory effect of GPS on LPS-induced chondrocyte inflammation. **A**–**B** Chondrocytes were treated with LPS (5 μg/ml) for 24 h and GPS (10, 20 and 40 μM) for another 24 h. IL-1β and PGE2 expression in culture supernatants was examined by ELISA. **C**–**D** q-PCR was used to determine IL-1β and COX2 mRNA expression levels in chondrocytes. *P < 0.05, ***P < 0.001

The ELISA results showed a significant increase in IL-1β and PGE2 in chondrocytes after LPS administration. IL-1β and PGE2 production in the supernatant in the 40 μM GPS-treated group was significantly lower than that in the model group (Fig. 3A, B). In addition, the mRNA expression of IL-1β and COX2 in chondrocytes in the model group was significantly increased compared to that in chondrocytes in the control group and significantly decreased after treatment with different concentrations of GPS (Fig. 3C, D).

### Effect of GPS on the production of MMP-13 and ADAMTS5 in LPS-induced chondrocytes

**Fig. 4:**
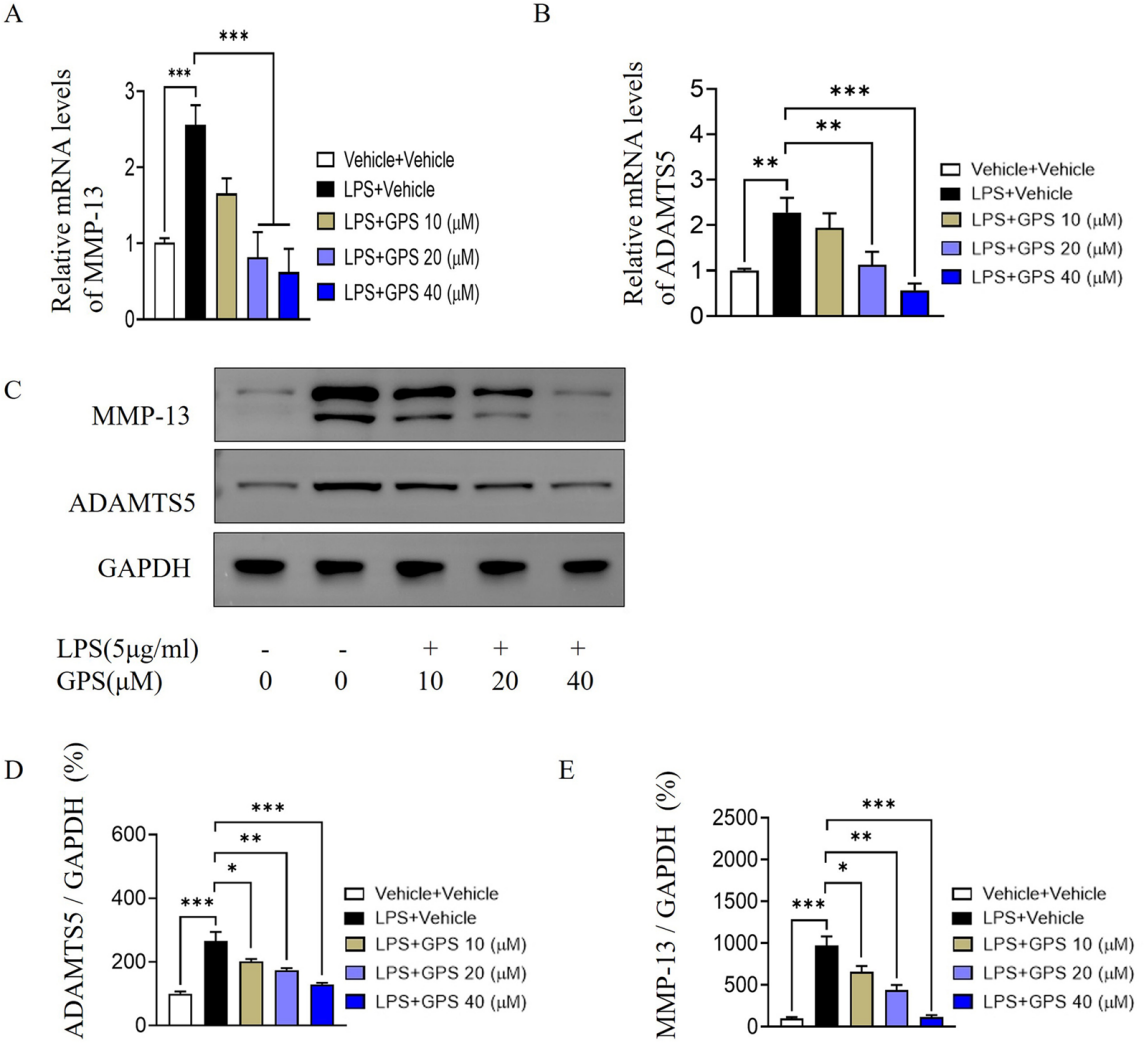
Effect of GPS on the LPS-induced expression of extracellular matrix-degrading proteases in chondrocytes. **A**–**B** The mRNA expression levels of MMP-13 and ADAMTS5 in chondrocytes were examined by q-PCR. **C** Western blot analysis of the protein expression of MMP-13 and ADAMTS5. **D**–**E** The relative expression levels of MMP-13 and ADAMTS5 were analyzed semiquantitatively. *P < 0.05, **P < 0.01, ***P < 0.001

Our results showed that 20 and 40 μM GPS significantly reduced the mRNA expression of MMP-13 and ADAMTS5 (Fig. 4A, B). Moreover, the protein levels of the MMP-13 and ADAMTS5 were significantly reduced compared to those in the model group (Fig. 4C–E).

### GPS reverses LPS-induced extracellular matrix degradation and chondrocyte hypertrophy

**Fig. 5:**
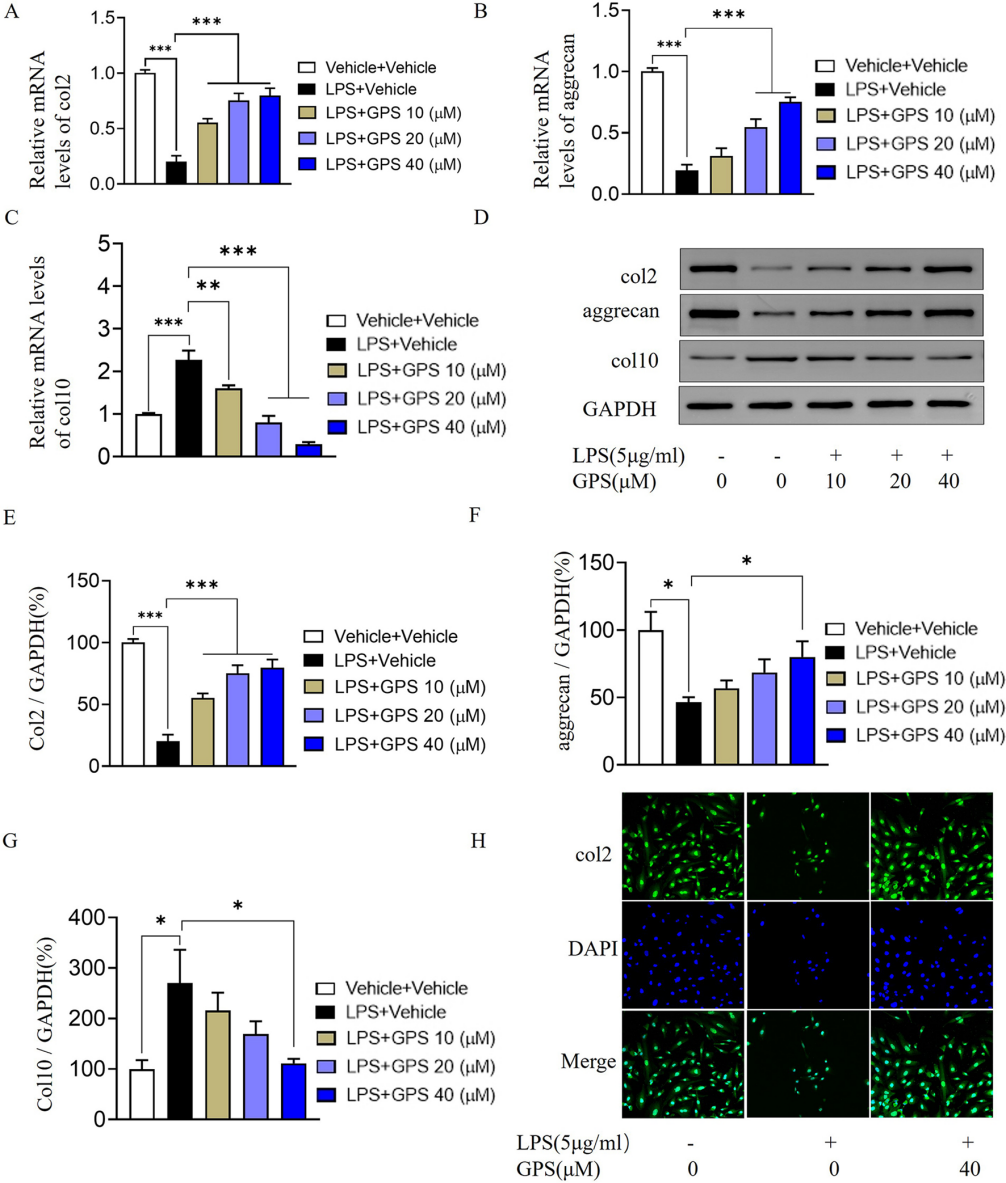
GPS inhibits chondrocyte extracellular matrix degradation and ameliorates chondrocyte hypertrophy. **A**–**C** The mRNA expression levels of col2, col10 and aggrecan in chondrocytes were examined by q-PCR. **D** Western blot analysis of the protein levels of col2, col10 and aggrecan in chondrocytes. **E**–**G** Relative expression levels of col2, col10 and aggrecan were analyzed semiquantitatively. **H** Immunofluorescence staining of col2. *P < 0.01, ***P < 0.001

LPS significantly decreased the mRNA expression of col2 and aggrecan, and the expression of col10 in chondrocytes was significantly increased. When different concentrations of GPS were applied, the mRNA expression of col2 and aggrecan significantly increased, and the mRNA expression of col10 significantly decreased (Fig. 5 A-C). In addition, Western blotting showed that the expression of col2 and aggrecan in chondrocytes in the model group was significantly decreased, and col10 expression was significantly increased. GPS (40 μM) significantly increased the expression of col2 and aggrecan in chondrocytes but decreased the expression of col10 (Fig. 5 D-G). Immunofluorescence staining revealed that GPS significantly inhibited col2 degradation (Fig. 5 H).

### GPS inhibits LPS-induced Stat3 activation

**Fig. 6:**
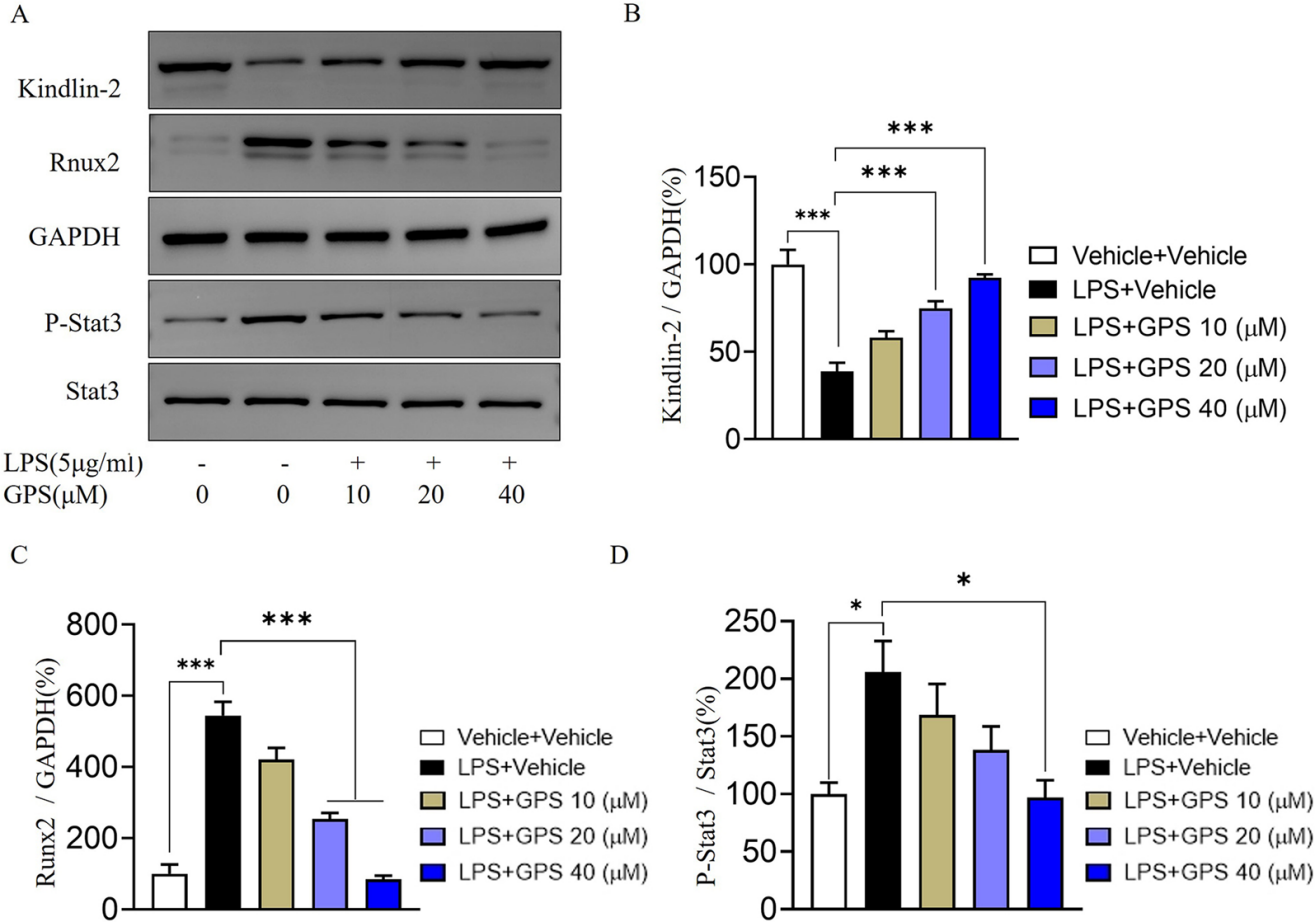
GPS reduces LPS-induced chondrocyte inflammation by inhibiting the Stat3/Runx2 signaling pathway. **A** Western blot analysis of the protein levels of Kindlin-2, Runx2, P-Stat3 and Stat3 in chondrocytes. **B**–**D** The relative expression levels of Kindlin-2, Runx2, and P-Stat were analyzed semiquantitatively. *P < 0.05, ***P < 0.001

To investigate the effect of GPS on the Stat3/Runx2 signaling pathway, the cells were incubated with 5 μg/ml LPS for 24 h and then treated with GPS (10, 20 and 40 μM) for 24 h. Western blotting showed that LPS significantly decreased the protein level of Kindlin-2 on the cell membrane surface, induced Stat phosphorylation and increased Runx2 expression. GPS treatment significantly reduced the degradation of the Kindlin-2 protein and inhibited Stat3 phosphorylation and Runx2 overproduction (Fig. 6).

## Discussion

With the aging of the population worldwide, the incidence of KOA is increasing. KOA is mainly caused by an imbalance between the destruction and repair of articular cartilage, and the degradation of cartilage extracellular matrix, the production of inflammatory mediators, and chondrocyte hypertrophy are involved in the pathological process of KOA [31]. Although NSAIDs are commonly used to treat OA, they only provide temporary relief from clinical symptoms and can cause significant side effects in the cardiovascular system, cerebrovascular system, and gastrointestinal tract [32]. Recently, there has been extensive interest in the use of GPS for the treatment of bone and joint diseases due to its potent anti-inflammatory and analgesic properties [19, 33, 34]. Our study revealed that the use of GPS effectively inhibited the inflammatory response in chondrocytes induced by LPS and alleviated hypertrophy. Consequently, it played a crucial role in protecting cartilage and establishing a novel theoretical foundation for the clinical use of traditional Chinese medicine monomer-based therapy.

The CCK-8 assay is commonly used to examine cell proliferation and cytotoxicity. Our findings revealed that chondrocyte viability was unaffected and that GPS at concentrations ranging from 0 to 40 μM had no cytotoxic effect on chondrocytes. Furthermore, 10, 20, and 40 μM GPS effectively prevented LPS-induced chondrocyte apoptosis and promoted chondrocyte proliferation. These results suggest that GPS can effectively reverse inflammation-induced chondrocyte apoptosis within a specific concentration range. Another study demonstrated that low concentrations of GPS were safe and nontoxic to chondrocytes and significantly inhibited inflammation in OA chondrocytes [26], which is consistent with our findings.

Inflammation plays a vital role in the progression of OA, and various inflammatory mediators contribute to its progression. IL-1β and PGE2 are particularly important in the development of OA [35, 36]. In this study, we used 5 μg/ml LPS to establish a cell model of osteoarthritis. Compared with those in the control group, the levels of IL-1β and PGE2 in the culture medium increased significantly after 24 h of exposure to LPS. Treatment of the osteoarthritis cell model with 40 μM gentiopicrin significantly decreased the production of PGE2 and the mRNA expression of COX2. These findings suggest that gentiopicrin inhibits the production of PGE2 by COX2 and reduces the inflammatory response of chondrocytes. However, further research is necessary to fully understand the underlying mechanisms.

Chondrocyte hypertrophy refers to an increase in chondrocyte volume, which is a critical step in the natural process of endochondral osteogenesis. However, abnormal activation of chondrocyte hypertrophy after injury and aging accelerates the pathological progression of OA [37]. Chondrocyte hypertrophy, which is characterized by increased expression of type X collagen (col10), Runx2, and MMP-13, is a significant factor in the development of OA [38]. The extracellular matrix of cartilage relies on type II collagen (col2) as its framework. Col2 works with aggrecan to maintain the structural integrity of cartilage and serves as a lubricant to aid in the mechanical support of cartilage [39]. The presence of CTX-II, which is a metabolite of col2, is easily detectable in urine and strongly correlates with the severity of KOA [40], suggesting that the loss of the extracellular matrix plays a crucial role in the pathogenesis of KOA. In our study, we treated chondrocytes with 5 μg/ml LPS and observed a significant decrease in the expression of col2 and aggrecan, and the expression of col10 increased significantly in the model group. However, different concentrations of GPS effectively reversed the degradation of the extracellular matrix in cartilage and reduced the expression of the chondrocyte hypertrophy marker col10. These findings suggested that GPS has a beneficial effect on chondrocytes and can reverse cellular hypertrophy.

Matrix metalloproteinases (MMPs) are a family of zinc-dependent proteolytic enzymes, and the expression of MMP-13, which can degrade col2, is most closely related to KOA [41]. Recombinant A disintegrin and metalloproteinase with thrombospondin (ADAMTS) is a protease that mediates the degradation of the ECM of chondrocytes, leading to the destruction of cartilage integrity and the degradation of aggrecan [42]. Even in the early stages of KOA, significant expression of ADAMTS5 was observed in articular cartilage [43]. Little et al. showed that compared with wild-type mice, OA model mice with whole-gene MMP-13 knockout exhibited relatively reduced articular cartilage degradation, whereas MMP-13 overexpression aggravated the progression of OA [44]. A recent study reported significant increases in MMP-3 and MMP-13 secretion in degenerative lumbar disc cartilage [45]. In our study, we observed an increase in the production of MMP-13 and ADAMTS5, as well as accelerated degradation of col2 in chondrocytes following LPS stimulation. However, the administration of GPS effectively blocked the LPS-induced upregulation of MMP-13 and ADAMTS5 and prevented extracellular matrix degradation. These findings suggest that GPS inhibits extracellular matrix degradation.

During the pathogenesis of KOA, the Stat3/Runx2 signaling pathway plays a role in ECM degradation, and the adhesion protein Kindlin-2 interacts with the cytoplasmic domain of integrin to activate integrin and regulate ECM adhesion and migration [46]. Previous studies on Kindlin-2 have mainly focused on bone development and the regulation of bone remodeling; however, its role in cartilage diseases has become a popular topic in recent years [47, 48]. According to a previous report, the absence of Kindlin-2 in articular chondrocytes in adult mice results in spontaneous OA and worsens surgery-induced OA lesions [49]. Western blotting showed that GPS significantly inhibited LPS-induced Stat3 phosphorylation in chondrocytes and reduced the acceleration of chondrocyte extracellular matrix catabolism caused by excessive Runx2 accumulation. Previous results have shown that GPS significantly inhibits Stat3 phosphorylation and ameliorates colon damage in mice with DSS-induced acute colitis [50], which is consistent with our results.

In summary, our study suggests that GPS reduces inflammation by inhibiting LPS-induced overproduction of IL-1β and PGE2 in chondrocytes, inhibits LPS-induced expression of MMP-13 and ADAMTS5 via the Stat3/Runx2 signaling pathway and reverses extracellular matrix degradation. It also reduces the production of col10, which is a marker of chondrocyte hypertrophy. These data indicate that GPS can inhibit the inflammatory response of KOA chondrocytes and chondrocyte hypertrophy to a certain extent, making it a promising drug for the treatment of KOA.

## Data Availability

The datasets supporting the conclusions of this article are all included within this article.

## Abbreviations

KOA: Knee osteoarthritis
LPS: Lipopolysaccharide
GPS: Gentiopicroside
CCK-8: Cell counting Kit-8
MMP-13: Matrix metalloproteinase-13
ADAMTS: A disintegrin and metalloproteinase with thrombospondin
GAPDH: Glyceraldehyde 3-phosphate dehydrogenase
ELISA: Enzyme-linked immunosorbent assay
EdU: 5-Ethynyl-2'-deoxyuridine
